# Association between homicide injury severity and benzodiazepine influence

**DOI:** 10.1080/20961790.2020.1767867

**Published:** 2020-06-03

**Authors:** Fredrik Tamsen, Joakim Sturup, Ingemar Thiblin

**Affiliations:** a Department of Surgical Sciences, Uppsala University, Uppsala, Sweden; b Swedish Police Authority, Stockholm, Sweden; c Department of Forensic Medicine, Swedish National Board of Forensic Medicine, Stockholm, Sweden

**Keywords:** Forensic sciences, forensic pathology, homicide, injury severity, injury severity score, quantification, benzodiazepines

## Abstract

There are case reports of offenders inflicting excessive injuries on their victims when under the influence of benzodiazepines. However, the potential association between benzodiazepine influence on the offender and victim injury severity in a general homicide population has not been studied. We investigated associations between offender positive testing for benzodiazepines or z-drugs (zolpidem, zopiclone and zaleplon) and victim injury severity. Data were drawn from 95 Swedish homicide cases from 2007–2009 in which offenders had known toxicology. There were no significant differences in injury severity between cases in which the offender tested positive *vs.* negative for benzodiazepines/z-drugs. Thus, the findings do not support the hypothesis that there is an association between benzodiazepine influence on the offender and victim injury severity in a general homicide population.

**Key points**
- Some previous studies have linked benzodiazepines to aggression, violence and excessive homicide injuries.
- The present study analysed the association between homicide injury severity and benzodiazepine status of the offender.
- Offenders who tested positive for benzodiazepines did not inflict more severe injuries on their victims.
- These findings do not support the hypothesis that benzodiazepine influence generally causes offenders to inflict more severe injuries on homicide victims.

Benzodiazepines are prescribed for various indications and are used as sedatives, anxiolytics and muscle relaxants. However, these drugs sometimes cause paradoxical reactions, including rage and aggressive behaviour [1]. One review of the relationship between different drugs and aggressive behaviour concluded that the perception of benzodiazepines is somewhat contradictory [2]. Some case reports have linked benzodiazepines with increased aggression, and experimental models on animals and humans have confirmed this link [3]. One proposed explanation for the association with aggression is that benzodiazepines may weaken empathy, although one experimental study did not find such a link with therapeutic doses of oxazepam [4]. Some case reports have observed not only aggressive but also sadistic violent behaviour in individuals under the influence of flunitrazepam [5]. Conversely, benzodiazepines are used by clinicians as an anti-aggression drug [2]. It is possible that this discrepancy is dose related, and that lower benzodiazepine doses are more often linked to aggression whereas higher doses are used in the management of aggression. However, contrary to this hypothesis, a study on the triggering effect of drugs on violent crimes found that large doses of benzodiazepines triggered interpersonal violence whereas therapeutic doses did not [6].

There are several ways to assess victim injury severity. For some purposes (e.g. when analysing bizarre behaviour and injuries), plain descriptions are probably the most useful [5, 7]. In contrast, well-defined variables are more useful in examinations of larger groups of homicide victims and when making comparisons between subgroups [8–11]. However, some variables and terms, such as “excessive wounding”, are still hard to define precisely. Such ambiguities have a negative effect on interrater reliability and complicate comparisons between studies. To make injury severity measurements more objective, some homicide studies have used injury severity scores, which are normally used in general trauma research [12–16]. Injury severity scores are derived from various methods that use a single number to quantify a person’s injuries.

One of the most commonly used scores in trauma research is the Injury Severity Score (ISS) [17]. The ISS is based on the Abbreviated Injury Scale (AIS), which is a consensus-driven document that contains almost all possible injuries and assigns them a score between 1 (least severe) and 6 (most severe) [18, 19]. To calculate the ISS, the body is divided into six regions. The highest AIS score in each of the three most severely injured regions are squared and added together. This sum constitutes the ISS. A modified ISS has also been developed, and is called the New ISS (NISS) [20]. In the NISS, the squares of the three highest AIS scores are summed, irrespective of body region. Both the ISS and NISS have been extensively used and validated in trauma research [21]. One advantage of using these measures in homicide research is that they are clearly defined, which helps to increase reliability. However, these scores are designed to predict morbidity and mortality; the purpose of assessing homicide victim injuries is often different. Most homicide studies seek to obtain an overall picture of the injuries or identify specific injury patterns [8, 22]. Even if a severity score has been validated for trauma victims, this validity may not transfer to homicide victims.

Other studies have used injury severity scores designed specifically for homicide victims [13–16, 23–25]. The Homicide Injury Scale (HIS) is a six-grade scale that takes into account both the cause of death and the severity of related injuries [25]. The Sum of AIS (SAIS) is calculated by adding together the AIS scores for all injuries sustained by a victim [14]. There are also variants of the SAIS in which only injuries to specific body parts are added, such as the SAIS face [13]. Using injury severity scores, previous studies have found changes in injury severity over time [16, 23] and associations between injury severity and the victim–offender relationship [13], as well as other homicide characteristics [25]. Thus, injury scores have proven to be useful in homicide research and facilitate a scientific approach to this aspect of criminology. The aim of the present study was to investigate possible associations between victim injury severity and positive *vs.* negative offender benzodiazepine toxicology. The pharmacodynamics and adverse effects of the so-called z-drugs (zolpidem, zopiclone and zaleplon) are similar to those of benzodiazepines [26] and so were also included. Drawing on previous literature and anecdotal evidence, our hypothesis was that a positive toxicology for benzodiazepines or z-drugs in offenders would be associated with more severe victim injuries.

## Materials and methods

### Study design and case identification

This was a retrospective, register-based study in which all victims (*n* = 273) and perpetrators (*n* = 257) of homicides in Sweden from January 1, 2007, to December 31, 2009, were considered for inclusion. Victims were identified from the case registry of the Swedish National Board of Forensic Medicine, which includes all individuals who have undergone a medicolegal autopsy in Sweden. The registry also includes the identification number of the police report, which was obtained together with court documents to access circumstantial data. Offenders were identified through the Swedish National Crime Register and linked to victims using court documents. The victim data included the autopsy protocol, sex and age. The offender data comprised sex, age and the presence or absence of benzodiazepines or z-drugs.

From a total of 273 victims, 63 were excluded: 28 owing to secondary trauma (fall from height, hit by vehicle, extensive burning, drowning, hypothermia), 28 owing to circumstances that made the injury assessment difficult (putrefaction and prolonged hospital care) and seven owing to other aggravating circumstances (e.g. incomplete protocol). This left *n* = 210 victims in the study. Associated with these victims were 99 offenders with toxicological data. Four offenders with multiple victims were excluded. Thus, 95 offenders and their associated victims (*n* = 91) were included in the study.

### Toxicological data

Blood specimens from the offenders were analysed using chromatography methods [27]. Results were included if the blood test had been obtained within 48 h of the homicide. The relevant drugs for the present study were the benzodiazepines diazepam, nordazepam, oxazepam, temazepam, lorazepam, alprazolam, nitrazepam, flunitrazepam, clonazepam and triazolam, as well as the benzodiazepine-related z-drugs zopiclone, zolpidem and zaleplon. The toxicological results were registered as showing either the presence or absence of any of these drugs.

### Injury severity measures

Victim injuries were scored using the HIS, ISS, NISS and SAIS. In addition to the whole body SAIS, we also calculated SAIS scores for the following body parts: head, face, neck, thorax, abdomen, arms and legs.

### Statistical analysis

Cases were subdivided into five groups according to the type of lethal injury: blunt, sharp, gunshot, asphyxia, and two or more types. A comparison of injury severity between victims with offenders that were positive *vs.* negative for benzodiazepines was performed using the Mann–Whitney *U* test. Data distribution was examined using multiple scatterplots. A *P*-value < 0.05 was considered statistically significant. Analyses were conducted using the statistical programme R (www.r-project.org).

## Results

Offender and victim sex and age are shown in Tables 1, respectively. Male offenders were on average 4 years younger than male victims, whereas the mean ages of female offenders and victims were approximately equal.

**Table 1.** Age and sex of offenders and victims (years, mean±SD).

	Males		Females		Total	
Subjects	*n*	Age	*n*	Age	*n*	Age
Offenders	87	35.3±14.4	8	40.5±18.1	95	35.7±14.7
Victims	55	39.1±16.2	36	40.8±18.3	91	39.7±17.0

**Table 2.** Presence or absence of benzodiazepines in offenders *vs.* trauma modality. Number of offenders (column/row percentages)^a^.

Benzodiazepines	Two or more	Blunt	Sharp	Gunshot	Asphyxia	Total
No	8 (73%/11%)	9 (82%/12%)	37 (73%/50%)	12 (92%/16%)	8 (89%/11%)	**74 (–/100%)**
Yes	3 (27%/14%)	2 (18%/10%)	14 (27%/67%)	1 (8%/5%)	1 (11%/5%)	**21 (–/100%)**
**Total**	**11 (100%/–)**	**11 (100%/–)**	**51 (100%/–)**	**13 (100%/–)**	**9 (100%/–)**	**95**

^a^ The percentages may not total 100 due to rounding.

Table 2 shows trauma modalities according to whether the offender tested positive or negative for benzodiazepines. In the benzodiazepine-positive group, the most common modalities were sharp force (67%) and multiple trauma (14%) types; gunshot (5%) and asphyxia (5%) were the least common modalities.

**Table 3.** *P*-values of Mann–Whitney *U* tests for differences in injury scores according to presence *vs.* absence of benzodiazepines in the offender.

Modality	HIS	ISS	NISS	SAIS	SAIS abdomen
Two or more	0.4795	0.6049	0.8372	1.0000	0.5428
Blunt	1.0000	0.9058	0.1229	0.2182	0.9051
Sharp	0.4873	0.3773	0.9746	0.8328	**0.0491**
Gunshot	1.0000	0.4913	0.8891	0.7890	0.6244
Asphyxia	0.3545	1.0000	0.6434	0.6667	0.2142

HIS: Homicide Injury Scale; ISS: Injury Severity Score; NISS: New Injury Severity Score; SAIS: Sum of Abbreviated Injury Scale. *P*-values < 0.05 indicate statistically significant differences in injury scores. Significant *P*-values are in bold.

**Figure 1.**
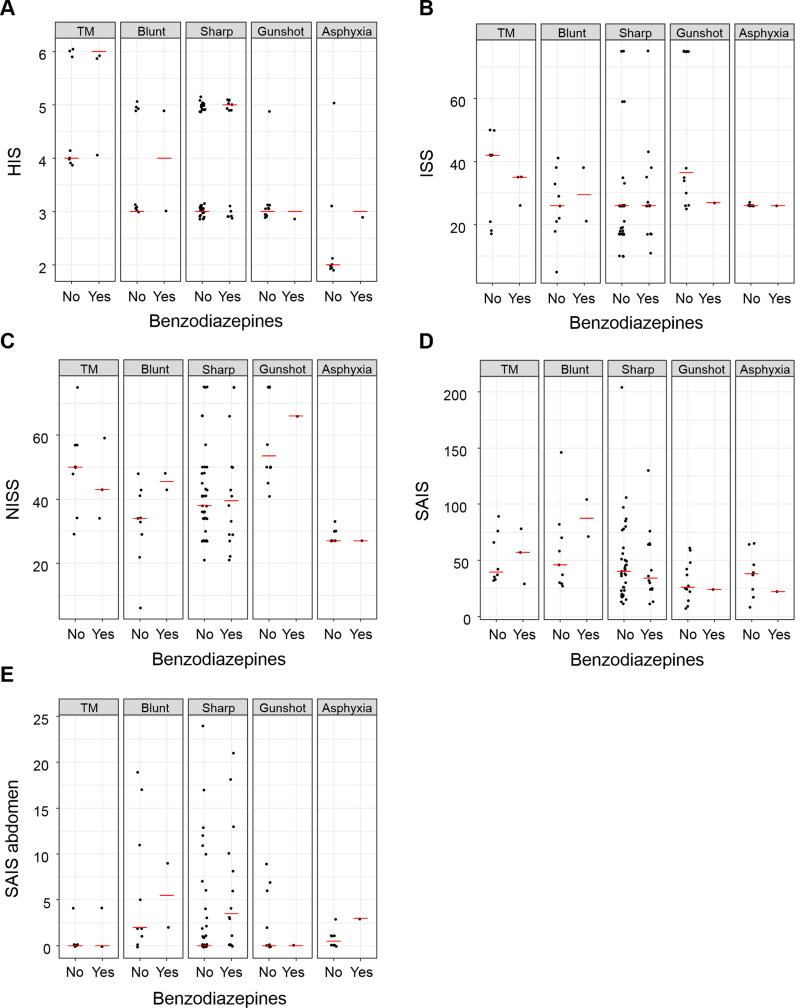
Homicide Injury Scale (HIS), Injury Severity Score (ISS), New Injury Severity Scale (NISS), Sum of Abbreviated Injury Scale (SAIS), and SAIS abdomen scores (A–E) by benzodiazepine influence and injury type for 95 Swedish homicide offenders and their victims. Dots indicate individual victims; red lines are median values. TM: two or more.

A selection of scatterplots generated for the 11 injury scores we used is shown in Figure 1. In the figures, the different boxes represent different trauma modalities. The dots in each box represent the scores of individual victims. This illustrates the distribution of injury scores according to whether the offender was positive *vs.* negative for benzodiazepines. Overall, these figures indicate that the injury scores were widely distributed within each modality, and there were no obvious differences between homicides in which the offender was positive *vs.* negative for benzodiazepines. In particular, there was no general tendency for benzodiazepine-positive offenders to inflict more injuries than benzodiazepine-negative offenders. On the contrary, for many of the cases that showed high SAIS values (>50–100), the offender tested negative for benzodiazepines (Figure 1D). This pattern held for all injury types. The same pattern can be seen for ISS and NISS scores (Figures 1B and 1C).

The only statistically significant association between injury severity and benzodiazepine influence was found for SAIS abdomen scores for deaths from sharp force (Table 3). In this group, victims of benzodiazepine-positive offenders had higher injury severity scores.

## Discussion

Overall, the scatterplots indicate that injury severity as measured in this study does not seem to be associated with the presence *vs.* absence of benzodiazepines in the offender. Only one of the comparisons was significant. Victims of offenders who were positive for benzodiazepines had a higher SAIS abdomen score when the lethal injury type was sharp force. However, the scatterplot shows a wide spread. In addition, many comparisons were performed, which increases the likelihood that some statistically significant differences occurred by chance. Therefore, this finding needs cautious interpretation and is not considered relevant.

The findings did not support our hypothesis that benzodiazepine influence in the offender would be associated with more severe injuries in the victim. However, there are documented cases in which benzodiazepines seem to have played a causal role in the extent and character of injuries [5]. The present authors have also, in their own work, seen cases with extensive injuries where the circumstances and testimonies suggest that benzodiazepine influence was a factor.

One possible reason for the lack of relevant correlations here is that benzodiazepines are important in the chain of events leading to excessive injuries in some cases, but that these cases are rare. Our results suggest that the amount and severity of injuries are not sufficient to separate benzodiazepine-intoxicated offenders from others at the group level. However, this does not exclude the possibility that more subjective injury assessment to identify abnormal injuries, such as sharp penetrating eye injuries, may uncover a link to benzodiazepine influence. The methods we used in this study are not useful for these types of distinctions. Another reason for our null finding may be that there is no link between benzodiazepines and injuries. A common pitfall in personal experience is confirmation bias. When a forensic pathologist or another individual working with homicides has a case with extensive or bizarre injuries, he/she automatically attempt to find an explanation. If there is proof that the offender was intoxicated with benzodiazepines, this is notable, as it is consistent with what the investigator already believes. If the offender was clean of benzodiazepines, the investigator may try harder to find an alternative explanation.

The study sample was small, so the findings need to be confirmed in future studies. However, the results indicate that there is at least no strong general connection between benzodiazepine influence and injury severity, as measured by standardised injury scores. One weakness of this study is the offender toxicological data. Drug test results were included if the tests were performed up to 48 h following the offence. Thus, they may not reflect the state of drug influence at the time of the homicide.

The diverging results from previous studies on whether or not benzodiazepines cause aggression may be because different benzodiazepines and z-drugs affect aggression and empathy in different ways. Our data contained no information about the type(s) of benzodiazepines or other drugs that each offender tested positive for. Benzodiazepines differ to some extent in both their pharmacodynamics and pharmacokinetics [28]. Because of this, the best way of investigating their potential role in homicides would be to analyse each type of benzodiazepine individually. By looking at them as a group, an effect in one type may be obscured by a lack of effect in other types. However, our dataset was too small to conduct this type of subanalysis and retain a reasonable level of statistical power. We believe that our analysis is a first step towards future studies with larger datasets in which specific benzodiazepines can be studied individually.

Another weakness is that we have no information about concentrations. This may be important, as previous studies have shown a tendency towards aggression only when there is a high consumption of benzodiazepines [6].

## Conclusion

There were no relevant associations between victim injury severity and the presence *vs.* absence of benzodiazepines or z-drugs in the offender’s blood in a general homicide population. Although the results do not support a connection between offender benzodiazepine influence and victim injury severity, there may be different settings were such a connection exists (e.g. specific types and amounts of benzodiazepines, specific types of injuries, and homicide subgroups).
